# Supplementary material for: Comparative genomics provides new insights into the diversity, physiology, and sexuality of the only industrially exploited tremellomycete: Phaffia rhodozyma
Source: BMC Genomics. 2016 Nov 9;17:901. doi: 10.1186/s12864-016-3244-7 (PMC5103461; doi:10.1186/s12864-016-3244-7)
Supplement: Additional file 6: — List of orphan genes with links to PFAM (related to Additional file 1: Table S1). (ZIP 1428 kb) [file 12864_2016_3244_MOESM6_ESM.zip › BLAST_HTML_FTR/G00358_P.html]

BLAST Search Results


```
BLASTP 2.2.27+


Reference:
Stephen F. Altschul, Thomas L. Madden, Alejandro A. Schäffer,
Jinghui Zhang, Zheng Zhang, Webb Miller, and David J. Lipman (1997),
"Gapped BLAST and PSI-BLAST: a new generation of protein database
search programs", Nucleic Acids Res. 25:3389-3402.


Reference for
composition-based statistics:
Alejandro A. Schäffer, L. Aravind, Thomas L. Madden, Sergei
Shavirin, John L. Spouge, Yuri I. Wolf, Eugene V. Koonin, and
Stephen F. Altschul (2001), "Improving the accuracy of PSI-BLAST
protein database searches with composition-based statistics and
other refinements", Nucleic Acids Res. 29:2994-3005.


Database: nr
           71,551,133 sequences; 26,053,659,533 total letters


Query= G00358_P

Length=870
                                                                      Score     E
Sequences producing significant alignments:                          (Bits)  Value

emb|CED83247.1|  hypothetical protein [Xanthophyllomyces dendrorh...  1372    0.0  


 >emb|CED83247.1| hypothetical protein [Xanthophyllomyces dendrorhous]
Length=864

 Score = 1372 bits (3550),  Expect = 0.0, Method: Compositional matrix adjust.
 Identities = 863/869 (99%), Positives = 863/869 (99%), Gaps = 5/869 (1%)

Query  1    MSAGRHAAGPLNPSTPIRKPTRPSSNLDTTTASSSPASPSSLTGSRTGQSSDDTPSKKAP  60
            MSAGRHAAGPLNPSTPIRKPTRPSSNLDTTTASSS     SLTGSRTGQSSDDTPSKKAP
Sbjct  1    MSAGRHAAGPLNPSTPIRKPTRPSSNLDTTTASSS-----SLTGSRTGQSSDDTPSKKAP  55

Query  61   SRGTVRLKRQPGAKGVVQYQSLEQLLAQAGYTETRVITPESIRIKERAAWIRAQEEEEQQ  120
            SRGTVRLKRQPGAKGVVQYQSLEQLLAQAGYTETRVITPESIRIKERAAWIRAQEEEEQQ
Sbjct  56   SRGTVRLKRQPGAKGVVQYQSLEQLLAQAGYTETRVITPESIRIKERAAWIRAQEEEEQQ  115

Query  121  EATHARETKEVSEIERFLFEDHFSSSTTDSTSQLDSNSDTDTDTVESIPKSEPGSGSTSL  180
            EATHARETKEVSEIERFLFEDHFSSSTTDSTSQLDSNSDTDTDTVESIPKSEPGSGSTSL
Sbjct  116  EATHARETKEVSEIERFLFEDHFSSSTTDSTSQLDSNSDTDTDTVESIPKSEPGSGSTSL  175

Query  181  GRNVDEQSERETGKAKKPWSAFRPVVDNRQHSRQHRHQQLHHRLSKEWDSSSNAIVYHYP  240
            GRNVDEQSERETGKAKKPWSAFRPVVDNRQHSRQHRHQQLHHRLSKEWDSSSNAIVYHYP
Sbjct  176  GRNVDEQSERETGKAKKPWSAFRPVVDNRQHSRQHRHQQLHHRLSKEWDSSSNAIVYHYP  235

Query  241  ADAFSLVDHPTTGRMPRAVPSFQDIQNGHNNNINNCHSEEECGSLSSSLSTFSAADTIIL  300
            ADAFSLVDHPTTGRMPRAVPSFQDIQNGHNNNINNCHSEEECGSLSSSLSTFSAADTIIL
Sbjct  236  ADAFSLVDHPTTGRMPRAVPSFQDIQNGHNNNINNCHSEEECGSLSSSLSTFSAADTIIL  295

Query  301  SPPDLNVQAFENQSASEDDADDDQSYATWSIPFNESVEDLRMSQASSRPIRPFNRSIHHG  360
            SPPDLNVQAFENQSASEDDADDDQSYATWSIPFNESVEDLRMSQASSRPIRPFNRSIHHG
Sbjct  296  SPPDLNVQAFENQSASEDDADDDQSYATWSIPFNESVEDLRMSQASSRPIRPFNRSIHHG  355

Query  361  QTPLPLPNAGPAQQALEFSESDVREDKSLFASSEAARSLRRISSLAQLSSPNSSASTSVK  420
            QTPLPLPNAGPAQQALEFSESDVREDKSLFASSEAARSLRRISSLAQLSSPNSSASTSVK
Sbjct  356  QTPLPLPNAGPAQQALEFSESDVREDKSLFASSEAARSLRRISSLAQLSSPNSSASTSVK  415

Query  421  PKFFNAFTFFSSSSFSPASDEAILKRPALSTQPKLPDQAHDTLLKRTLRHTASTPVLARR  480
            PKFFNAFTFFSSSSFSPASDEAILKRPALSTQPKLPDQAHDTLLKRTLRHTASTPVLARR
Sbjct  416  PKFFNAFTFFSSSSFSPASDEAILKRPALSTQPKLPDQAHDTLLKRTLRHTASTPVLARR  475

Query  481  TGSSNSSSEAVPPLPTGWLETIKNTLQSTISSTPSSIIPSTSTLMPKDAPPILRQSSSPA  540
            TGSSNSSSEAVPPLPTGWLETIKNTLQSTISSTPSSIIPSTSTLMPKDAPPILRQSSSPA
Sbjct  476  TGSSNSSSEAVPPLPTGWLETIKNTLQSTISSTPSSIIPSTSTLMPKDAPPILRQSSSPA  535

Query  541  VLSPPLSSSSASCSENVANEKPVSVHLRSAPLIPKLTPSTVVCGSEEGEDLPPFSSLSFF  600
            VLS PLSSSSASCSENVANEKPVSVHLRSAPLIPKLTPSTVVCGSEEGEDLPPFSSLSFF
Sbjct  536  VLSFPLSSSSASCSENVANEKPVSVHLRSAPLIPKLTPSTVVCGSEEGEDLPPFSSLSFF  595

Query  601  NQSSGSKSGSTSTSSPVTPHGSVSETIPSPVLTPKISFGEGEGWSRTTCPGWVWTEGTDD  660
            NQSSGSKSGSTSTSSPVTPHGSVSETIPSPVLTPKISFGEGEGWSRTTCPGWVWTEGTDD
Sbjct  596  NQSSGSKSGSTSTSSPVTPHGSVSETIPSPVLTPKISFGEGEGWSRTTCPGWVWTEGTDD  655

Query  661  REEDGWYATPMCSFEDSPLDNSSPPRFRAATVSSSSSSFTTSPASTASATSAATGGSRFG  720
            REEDGWYATPMCSFEDSPLDNSSPPRFRAATVSSSSSSFTTSPASTASATSAATGGSRFG
Sbjct  656  REEDGWYATPMCSFEDSPLDNSSPPRFRAATVSSSSSSFTTSPASTASATSAATGGSRFG  715

Query  721  ILRAQRSIKSLRETLAADSTAQAVALAKAKAQAEIKAAAAAAVAAATAANHAFRFKTPNR  780
            ILRAQRSIKSLRETLAADSTAQAVALAKAKAQAEIKAAAAAAVAAATAANHAFRFKTPNR
Sbjct  716  ILRAQRSIKSLRETLAADSTAQAVALAKAKAQAEIKAAAAAAVAAATAANHAFRFKTPNR  775

Query  781  RQNKASSPLSIKHRSGPVPIFCISSPDTLELGLPPRELDVEEEERRASALVALALSSSRK  840
            RQNKASSPLSIKHRSGPVPIFCISSPDTLELGLPPRELDVEEEERRASALVALALSSSRK
Sbjct  776  RQNKASSPLSIKHRSGPVPIFCISSPDTLELGLPPRELDVEEEERRASALVALALSSSRK  835

Query  841  EAEDEGWGGGRRKAVRKERKVRREVGRKK  869
            EAEDEGWGGGRRKAVRKERKVRREVGRKK
Sbjct  836  EAEDEGWGGGRRKAVRKERKVRREVGRKK  864


Lambda      K        H        a         alpha
   0.308    0.122    0.344    0.792     4.96 

Gapped
Lambda      K        H        a         alpha    sigma
   0.267   0.0410    0.140     1.90     42.6     43.6 

Effective search space used: 10369889559630


  Database: nr
    Posted date:  Sep 23, 2015 12:05 AM
  Number of letters in database: 26,053,659,533
  Number of sequences in database:  71,551,133


Matrix: BLOSUM62
Gap Penalties: Existence: 11, Extension: 1
Neighboring words threshold: 11
Window for multiple hits: 40
```
